# A Mechanistic Model for Naive CD4 T Cell Homeostasis in Healthy Adults and Children

**DOI:** 10.3389/fimmu.2013.00366

**Published:** 2013-11-11

**Authors:** Tharindi Hapuarachchi, Joanna Lewis, Robin E. Callard

**Affiliations:** ^1^Institute of Child Health and CoMPLEX, University College London, London, UK

**Keywords:** naive T cells, homeostasis, CD4 T cells, mathematical modeling, mechanistic modeling, children

## Abstract

The size and composition of the T lymphocyte compartment is subject to strict homeostatic regulation and is remarkably stable throughout life in spite of variable dynamics in cell production and death during T cell development and immune responses. Homeostasis is achieved by careful orchestration of lymphocyte survival and cell division. New T cells are generated from the thymus and the number of peripheral T cells is regulated by controlling survival and proliferation. How these processes combine is however very complex. Thymic output increases in the first year of life and then decreases but is crucial for establishing repertoire diversity. Proliferation of new naive T cells plays a crucial role for maintaining numbers but at a potential cost to TCR repertoire diversity. A mechanistic two-compartment model of T cell homeostasis is described here that includes specific terms for thymic output, cell proliferation, and cell death of both resting and dividing cells. The model successfully predicts the homeostatic set point for T cells in adults and identifies variables that determine the total number of T cells. It also accurately predicts T cell numbers in children in early life despite rapid changes in thymic output and growth over this period.

## Introduction

The naive T cell compartment in humans is generated early in development by the thymus and then maintained throughout life by continued export from the thymus and cell division in the periphery. In adult humans, the naive T cell compartment is comprised of roughly 10^11^ cells circulating between the blood and the peripheral lymphoid organs. It is estimated to comprise at least 10^8^ different T cell receptor specificities (1) providing a broad spectrum of protection in a diverse pathogen environment. The size and composition (T cell receptor diversity) of the naive T cell compartment are subject to strict homeostatic regulation and are remarkably stable throughout adult life despite changing rates of cell production and death during T cell development and immune responses (2, 3). Homeostasis is achieved by control of lymphocyte survival and cell division. Naive T cell survival and peripheral cell division depends on access to the cytokine IL7 (4–7) and TCR signals (8, 9) through contact with self-peptide MHC (spMHC) on dendritic cells (10). In lymphoreplete mice, naive T cells are largely non-cycling (11) whereas homeostatic cell division plays an important role in maintaining naive T cell homeostasis in humans, where cell division is evident in the naive pool (12, 13).

**Figure 1:**
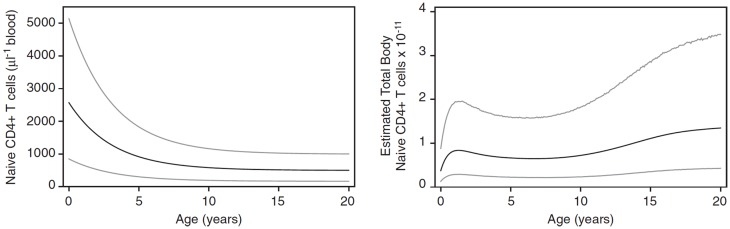
**Changes in naive CD4 T cell concentration and total whole body numbers with age**. Taken from Bains et al. (14).

In children, homeostatic control of the T cell compartment may be affected by both the growth of the child with the accompanying increased blood volume (14) and changes in thymic output, which increases to a maximum over the first year of life and then declines to reach an approximately steady level by the age of about 20 years (15). As a result, the CD4 naive T cell count (cells/μl) in children declines over the first 10–20 years of life whereas the total number of naive CD4 cells increases as the child grows (Figure 1). This raises important questions about whether the homeostatic mechanisms themselves change during early life or whether the numbers of naive CD4 T cells observed are determined only by the changes in thymic output and growth.

To date, our understanding of the processes controlling survival and proliferation of T cells has been largely qualitative and detailed quantitative knowledge of how homeostatic responses result in the observed equilibrium of the T cell pool with a given size and composition is lacking. Here, a two-compartment mathematical model of homeostasis is presented incorporating specific terms for thymic export into the naive CD4 compartment, rates of entry into cell division and death (survival) rates for both the resting and dividing cell compartments. In this sense, the model can be considered as mechanistic in comparison to empirical or descriptive models where the parameters have no direct biological meaning. The results illustrate the importance of T cell dynamics for the maintenance of constant naive CD4 T cell numbers in adults and the growth of the T cell compartment in children.

## Materials and Methods

### A model of naive T cell homeostasis

**Figure 2:**
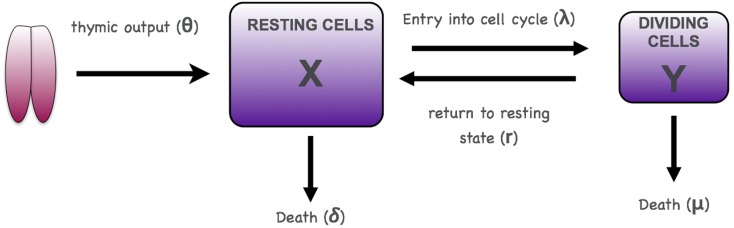
**Scheme for two-compartment model of homeostasis**.

T cell homeostasis can be described using a two-compartment model of resting and dividing cells with input from the thymus into the resting compartment as shown in Figure 2 (16, 17). In this model we will consider only naive CD4 T cells assuming no antigenic stimulation and maturation of naive to memory cells. The same model could in principle also be applied to memory cells and CD8 T cells.

This model can be expressed mathematically using two coupled ordinary non-linear differential equations:

$$\begin{array}{llll} & \frac{dX}{dt}=\theta +2rY-\lambda_{\left(X+Y\right)}X-\delta_{\left(X+Y\right)}X\; & & \;\\ & \frac{dY}{dt}=\lambda_{\left(X+Y\right)}X-rY-\mu_{\left(Y\right)}\; & & \; \end{array}$$

where *X* is the number of non-dividing (resting) T cells and *Y* the number of cells undergoing cell division (Figure 2). The parameter θ represents T cell export from the thymus, λ the rate at which resting cells enter cell division, r the rate at which dividing cells return to the resting state, δ the death rate of resting cells, and μ the death rate of dividing cells. λ, r, δ, and μ are all first order rate constants, in units of day^−1^, whereas θ is a zero-order constant, in units of cells day^−1^.

To develop this model, it is important to have biologically appropriate forms for each of these parameters. Thymic output is known to vary with age with a maximum at about 1 year, which then declines rapidly until about 20 years of age and more slowly thereafter (15, 18). The value of θ for a 20 year old has been estimated to be 3 × 10^8^ CD4 T cells day^−1^ (15). This value was used for modeling CD4 T cell homeostasis in a young adult. In children, the value of θ changes rapidly with age. An expression for θ from 0 to 20 years was determined as described previously (15). An appropriate form for the rate of entry into cell cycle λ is (19)

$$\lambda =\lambda_{0}\exp\left[-N\left(t\right)∕\varepsilon \;\right]$$

where *N*(*t*) = *X*(*t*) + *Y* (*t*), i.e., the total number of T cells at time *t*.

This expression is based on competition between resting naive T cells for signals to enter cell division: TCR signaling by self-peptide MHC and resources such as IL7 (4, 5, 8, 9, 20, 21). The term λ_0_ represents the intrinsic ability of a T cell to respond under conditions of no competition (very few cells or an unlimited supply of homeostatic proliferative signals such as IL7), ε is proportional to the amount of resource (IL7) available and N is the total number of T cells competing for the resource. The rate of entry into cell cycle therefore deceases exponentially with decreasing resource or increasing cell number. The rate at which dividing cells return to the resting state r is determined by the length of time taken for one division [known to be about 6 h (19)] and experimental evidence that in homeostatic cell division cells return to the resting state after one division (19, 22). The death rate μ of activated T cells takes the form μ = μ′*Y*, which represents density-dependent AICD (activated induced cell death) by Fas–Fas ligand interactions (23, 24). Finally, the death rate of resting cells δ takes the form

$$\delta =\delta_{0}\exp\left[N\left(t\right)∕\rho \;\right].$$

Similar to λ, this term is also derived from the reported dependence of cell survival on competition for a survival signal such as IL7 (resource) where δ_0_ is the intrinsic ability of a cell to die under conditions of no competition (very few cells or an unlimited supply of the survival signal) and ρ is proportional to the amount of available resource providing the survival signal (IL7) (21). Parameter values used in the model are shown in Table 1.

**Table 1:** **Parameter values used for the model**.

Parameter	Description	Value
Θ	Thymic output for adult 20 years old	3 × 10^8^ cells day^−1^ (15)
λ_0_	Rate of entry into cell cycle with infinite resource	0.055 cell^−1^day^−1^
ε	Resource for entry into cell cycle	1
δ_0_	Death rate of resting cells with infinite resource	0.02 cell^−1^day^−1^
ρ	Resource for resting cell survival	100
r	Rate of return from dividing to resting state	4 day^−1^ (every 6 h)
μ	Death rate of dividing cells	15 day^−1^

The model was solved numerically using NDSolve, the proprietary numerical ODE solver in Mathematica that automatically selects the most appropriate method and adapts the step size so that the estimated errors are within the specified tolerance.

## Results

### Homeostatic set point in adults

**Figure 3:**
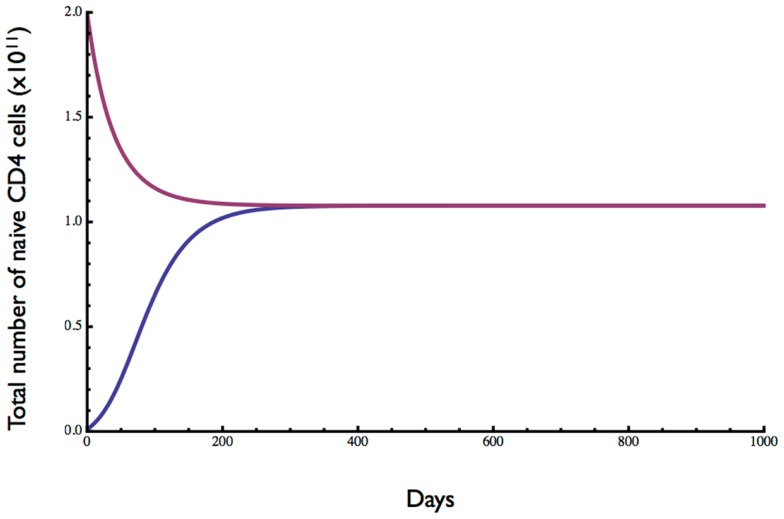
**Dynamics of naive CD4 T cell homeostasis in adults predicted by the model**. Starting with 0.01- or 2-fold the approximate number of naive CD4 T cells in a replete young adult, an equilibrium of about 10^11^ cells is reached within 200–300 days. Parameter values are given in Table 1.

The homeostatic set point for adults was examined by testing the behavior of the model starting with cell numbers well below and above the equilibrium and with an adult thymic output of 3 × 10^8^ cells day^−1^ (15). Initial conditions were 0 dividing cells and either 0.01 or 2 (×10^11^) resting cells. As shown in Figure 3, a stable equilibrium of total naïve CD4 T cell numbers (resting plus dividing) was obtained at just over 10^11^ cells, after 200–300 days (see also Figure 1). A Jacobian analysis showed that the solutions were stable over a wide range of parameter values for r (>0.281), μ′ (<106.79), ε (<1.01), and λ_0_ (>1.05 × 10^−14^) and stability did not depend on thymic output (θ), δ_0_, or ρ.

**Figure 4:**
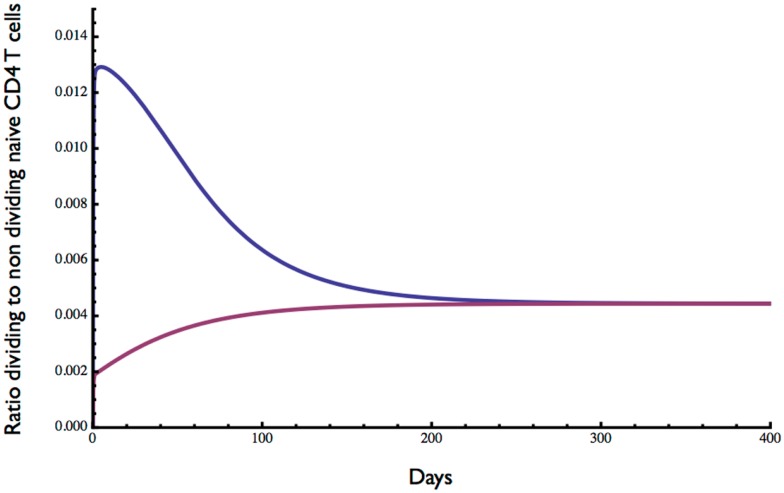
**Ratio of dividing to resting cells predicted by the model**. Starting with 0.01 × 10^11^ (blue curve) or 2 × 10^11^ (red curve) the approximate ratio of dividing to resting cells changes over time to reach an equilibrium of about 0.4%. As expected, the proportion of proliferating cells is greater when the initial cell number is low. Parameter values used in the model are the same as in Figure 3.

The ratio of dividing to resting cells is shown in Figure 4. With lymphopenic starting conditions of 0.01 (×10^11^) resting cells, the proportion of proliferating cells (blue curve) increased very rapidly from 0 to 0.013 and then slowly declined over about 200 days to reach an equilibrium at about 0.5%. In contrast, under starting conditions of excessive T cells, the ratio of dividing to non-dividing cells increased rapidly at first from 0 to about 0.2% and then slowly to reach the same equilibrium of about 0.5%. This equilibrium point is consistent with a low level of cell division in the naive compartment of adult humans as reported previously (25, 26).

### Effects of competition for survival and division signals

**Figure 5:**
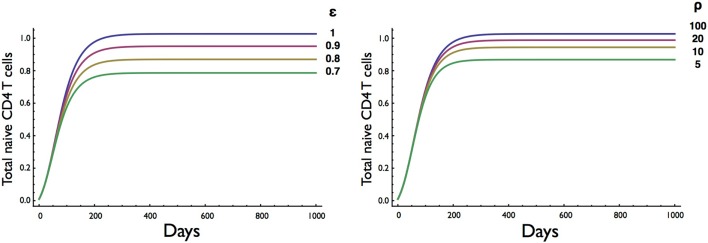
**Effect on T cell dynamics of changes in resource concentration for entry into cell division (ε) and survival (ρ)**. Other parameters are as in Table 1. It is noteworthy that the homeostatic equilibrium is more sensitive to changes in the resource parameter (ε) for entry into cell division than the parameter (ρ) for survival.

Next, we investigated the effect of the amount of resource available for cell division (ε) and cell survival (ρ) (Figure 5). Consistent with competition between naive CD4 T cells for a resource such as spMHC and/or IL7 in order to survive and undergo cell division, the number of cells at homeostatic equilibrium decreased as the resource terms ε for proliferation and ρ for survival decreased. Interestingly, the rate of entry into cell cycle was significantly more sensitive than survival to changes in resource concentration, consistent with different thresholds for proliferation and survival as previously described (27).

### T cell homeostasis in children

**Figure 6:**
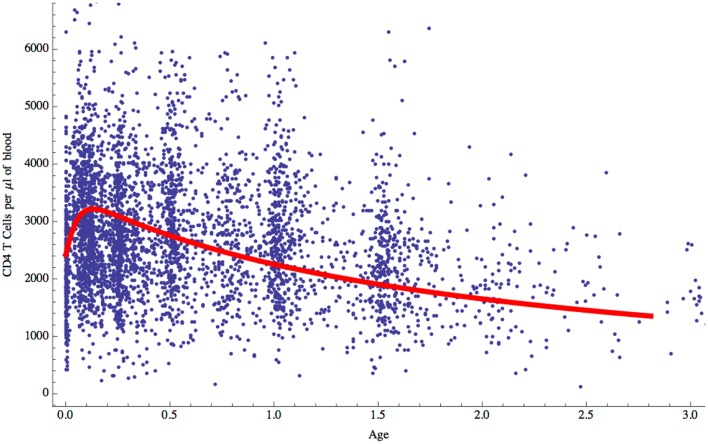
**Naive CD4 T cell concentrations (cells/μl of blood) predicted by the model for children aged 0–3 years (red curve) compared with clinical data for normal children**.

Having established the behavior of the two-compartment model for naive CD4 T cell homeostasis in adults, we sought to determine whether it could also be used to explain the changes in T cell numbers that occur during childhood. During the first few years of life as thymic output changes and children grow, the naive CD4 T cell concentration decreases while the total number increases (Figure 1). The question is whether the homeostatic mechanism described by the model is in itself enough to explain these variations, or whether different and changing mechanisms apply in children. To examine this question, the model was used to predict the concentration of naive CD4 T cells in cells/μl of blood by converting total numbers to concentration using the estimated blood volume of children at different ages (14). In addition, the changes in thymic output that occur over the first few years of life with a peak at 1 year and then a decline (15) were incorporated into the model. The prediction from the model was then simply compared without parameter fitting to data collected from a cohort of healthy children (born to HIV infected mothers) from the European Collaborative Study on HIV infected pregnant women and their children (28) (Figure 6). As can be seen, the model predicted the concentration of T cells over the first 3 years of life extremely well suggesting that the homeostatic mechanisms in children and adults are essentially the same with the only difference being thymic output and growth with a concomitant increase in blood volume.

## Discussion

The two-compartment mathematical model presented here is based on the known biology of naive T cell homeostasis. It is derived from an earlier simple model that ignored thymic output and competition for resources (17, 24). Although only naive CD4 T cells are considered here, the same model would essentially be applicable to CD8 T cells. Naive single positive CD4 T cells enter the peripheral pool from the thymus at rates ranging from 4 × 10^8^ to 2 × 10^9^ day^−1^ depending on age from 0 to 20 years, with a peak of 2 × 10^9^ day^−1^ at about 1 year of age (15). In addition to thymic output, maintenance of the naive T cell pool in humans also depends on peripheral T cell division (13). Naive T cell entry into cell division occurs in response to TCR signaling by self-peptide MHC and signals provided by IL7 (4–9, 29). Competition for these resources determines the rate of entry (19, 29). Similarly, survival depends on signaling by IL7 albeit at a different concentration threshold than for proliferation (27). The rate of exit from the cell cycle was taken from the approximate time taken to complete cell division [6 h (19)] and the death rate of the cells in cycle was modeled by the described Fas/Fas ligand mechanism of activated cells (24).

The mathematical model described here consists of two coupled non-linear differential equations representing resting and dividing cell compartments with parameters for thymic export, entry into cell division, return to the resting state and death (at different rates) of resting and dividing cells. Exponential forms were used for entry into cell division and death of cells in the resting compartment to represent the known competition for resources for cell proliferation and survival. Alternative functional forms for density dependence may be worth exploring in the future. The model is a mechanistic model based on the known biology of naïve T cell homeostasis so that the different parameters all have a biological interpretation as indicated in the methods. An alternative but non-mechanistic mathematical model of T cell homeostasis has been described (30), which depends on assumptions about the inheritability of life spans and it cannot therefore be easily compared to the model we describe here. Rather, in our model proliferation and death rates depend on competition for resources as supported by experimental evidence. Our model does however assume the naïve T cell population is homogeneous without taking into account clonal diversity and it would be of interest in the future to develop stochastic ODEs or agent based models.

When T cell export from the thymus was kept constant to represent a young adult, simulated T cell numbers converged from either low or high initial levels to a stable homeostatic equilibrium consistent with cell numbers in a normal, healthy adult. This is concurrent with the increase in T cells observed in response to lymphopenia and the decrease following T cell expansion after infection (16, 17). Consistent with previous studies, the death rate of proliferating cells is higher than that of resting cells in our model (31). The death rate of resting cells found here also agrees approximately with experimental results (32–34). The average time between cell divisions was about 50 days compared to 60 days in the model described by Yates (31). Another interesting aspect of these results was the interdivision time of cells, calculated to be around 30 days. This is comparable to the results of deuterium labeling experiments, which suggest an average of 26 days (16, 17, 34, 35).

The results obtained by altering parameter values gave a clear indication of the effect of the different rates of cell death and proliferation. The corresponding expected rise and fall in the set point of the T cell pool was reassuring. This set point appeared to be more sensitive to increments in the death rate of resting cells than to increases of the same order in the activation rate. Importantly, the T cell numbers at equilibrium decreased as the resource term for entry into cell division (ε) or the resource term for rescue from cell death (ρ) decreased although the equilibrium was less sensitive to changes in the resource required for survival (Figure 5). The sensitivity of the homeostatic T cell equilibrium to a resource, such as IL7, is potentially important for understanding conditions resulting in reduced CD4 T cell numbers, such as HIV, and the degree of recovery after treatment with antiretroviral therapy (ART). In a recent study, the degree of CD4 T cell recovery in children on ART was correlated with the initial (pre-ART) CD4 T cell count and the length of time between infection (at birth) and the commencement of treatment (36). One explanation for this finding is that HIV infection compromises lymph node structure and hence the ability to provide resources required for homeostatic T cell division (37, 38). The two-compartment model could then be a valuable tool for exploring T cell homeostasis in HIV and other conditions such as T cell reconstitution following stem cell transplantation.

The other question addressed by the model was whether the incorporated biological mechanisms were in themselves sufficient to explain the known decrease in naive CD4 T cell concentration over the first few years of life when T cell export from the thymus increases to a maximum at 1 year of age and then declines, and the child is growing in size with an accompanying increase in blood volume (Figure 1). Total naive CD4 T cell numbers obtained from the model were converted into T cell concentration in the blood using blood volume/age data (14). The model’s predictions were found to agree very well with real data from a cohort of children aged 0–3 years (Figure 6): the two-compartment model was able to reproduce the initial rise and subsequent slow decline in T cell count observed in healthy individuals over 0–3 years. These findings suggest that the changes in CD4 T cell counts in young children can be explained simply by the change in thymic output and body size as they grow and does not require any additional developmental changes to homeostatic mechanisms. It is important to point out that the thymic export model does not take memory cells into account. However, the proportion of memory cells in the CD4+ T cell pool in children is relatively small and therefore should not have a significant effect on these results (39).

In conclusion, we have presented a mechanistic two-compartment model of naive T cell homeostasis based on the known biology, which reproduces results obtained by other methods with good accuracy. It is likely to be an appropriate model for investigations of T cell reconstitution and homeostasis in diseases such as HIV, in patients given bone marrow transplantation and even for understanding reconstitution after thymic transplants for athymic patients with DiGeorge syndrome.

## Conflict of Interest Statement

The authors declare that the research was conducted in the absence of any commercial or financial relationships that could be construed as a potential conflict of interest.
